# 3D Visualization System in Descemet Membrane Endothelial Keratoplasty (DMEK): A Six-Month Comparison with Conventional Microscope

**DOI:** 10.3390/jcm11154312

**Published:** 2022-07-25

**Authors:** Alberto Morelli, Rosangela Ferrandina, Eleonora Favuzza, Michela Cennamo, Rita Mencucci

**Affiliations:** 1 Eye Clinic, Careggi Hospital, Department of Neurosciences, Psychology, Pharmacology and Child Health (NEUROFARBA), University of Florence, 50134 Florence, Italy; alberto.morelli@unifi.it (A.M.); elefavuzza@gmail.com (E.F.); michelacennamo@libero.it (M.C.); 2 Department of Biotechnology and Medical-Surgical Sciences, ‘Sapienza’ University of Rome, 04100 Latina, Italy; rosangela.ferrandina@gmail.com

**Keywords:** heads-up surgery, cornea, DMEK, graft surgery, 3D surgery, corneal densitometry

## Abstract

Background: To compare the efficacy and safety of Descemet membrane endothelial keratoplasty (DMEK) surgery using the three-dimensional (3D) display system NGENUITY to DMEK surgery performed with the traditional microscope (TM) in patients affected by Fuchs Endothelial Corneal Disease (FECD). Methods: Retrospective comparative study of 40 pseudophakic eyes of 40 patients affected by FECD who underwent DMEK surgery. Twenty patients (3D group) were operated on using the 3D display system and 20 patients (TM group) were operated on using the traditional microscope. Best spectacle corrected visual acuity (BSCVA), central corneal thickness (CCT), endothelial cell density (ECD) and corneal densitometry (CD) values were documented before and at 1, 3 and 6 months after DMEK. Intra- and postoperative complications were recorded. Results: The baseline assessments did not differ between the two groups (*p* > 0.05). Global surgical time and time to perform descemetorhexis were significantly lower in the TM group (*p* = 0.04 and *p* = 0.02, respectively). BSCVA, CCT, ECD and CD values did not differ significantly in the two groups at all follow-ups (*p* > 0.05). Complication rate was similar between the two groups. Conclusion: Three-dimensional display systems can be securely employed in DMEK surgery considering the satisfactory clinical outcomes, including Scheimpflug CD. Nevertheless, the slightly longer surgical time of the 3D DMEKs may lead to surgeons’ hesitancy. The main advantages of the heads-up approach may be the improved ergonomic comfort during surgery and the utility of assistants in surgical training.

## 1. Introduction

Endothelial keratoplasty (EK) is the surgical procedure of choice for the treatment of corneal decompensation associated with Fuchs Endothelial Corneal Dystrophy (FECD) [1,2]. Descemet membrane endothelial keratoplasty (DMEK), compared with other EK procedures, shows several advantages, including rapid visual recovery, better anatomical restoration due to the reduced graft thickness (10–15 µm) and minimal light scatter due to minimal interface irregularity [3].

Scheimpflug corneal densitometry (CD) is an objective method for accurately calculating corneal backscatter for defined concentric zones, thus providing an objective measurement for corneal transparency that is widely used after collagen cross-linking and after refractive surgery [4,5]. CD may also provide a feasible and objective method for monitoring corneal transparency after endothelial keratoplasty [6].

DMEK procedures have always been performed using traditional surgical microscopes, but, more recently, 3D visualization systems have also been employed. The term “heads-up” refers to the surgical procedures performed by viewing the 3D microscopic image on a panel display, providing a more natural and ergonomic posture for the surgeon [7]. One of the most used 3D visualization systems in ophthalmology is the NGENUITY 3D visualization system (Alcon, Forth Worth, TX, USA), which was used in our study. It is a modular system that is attached to the traditional microscope, which allows the surgeon to view a 3D stereoscopic image on a panel display using polarized glasses instead of looking at the eyepieces of the microscope.

Such 3D systems were originally employed for vitreoretinal surgery [8,9,10], but afterwards, their use was extended to anterior segment surgery, especially cataract and corneal graft surgery [11,12,13]. The use of heads-up procedures in cataract surgery was first described by Weinstock et al., who presented a retrospective analysis comparing surgeries performed using a standard binocular microscope versus a microscope equipped with a 3D visualization system. Excellent results were reported in both groups, with a minimal difference in total surgical time [14]. The use of heads-up in DMEK surgery was described for the first time by Galvis et al., who reported a case of a 68-year-old female with pseudophakic bullous keratopathy [12]. More recently, in 2020, a prospective, single-center, cross-sectional study was conducted at the Rothschild Foundation, Paris, France by Panthier et al. [13]. The study compared DMEK surgeries performed using a standard binocular microscope and the NGENUITY 3D visualization system. Each group included 12 cases: six single DMEK and six combined DMEK and cataract procedures. The authors reported that DMEK using a 3D display system was feasible, but it was more challenging and the total surgical time recorded was longer. However, it was considered certainly useful for instructional courses.

Only a few studies have evaluated the use of 3D systems in endothelial keratoplasty, and, to our knowledge, there are currently no studies in the literature examining corneal densitometry in patients who underwent heads-up DMEK surgery. The purpose of this study was to examine the surgical times, safety and clinical outcomes, including corneal densitometry, of DMEK surgery performed with a 3D system versus a traditional microscope with a six-month follow-up.

## 2. Materials and Methods

### 2.1. Design

This single-center, retrospective, controlled study included 40 eyes of 40 patients affected by Fuchs Endothelial Corneal dystrophy (FECD) who underwent DMEK surgery. Procedures were consecutively performed between 1 November 2019 and 28 February 2021 at the Azienda Ospedaliero-Universitaria Careggi, University of Florence, Florence, Italy.

Twenty DMEKs were consecutively performed using the NGENUITY 3D visualization system (3D group) and the other 20 DMEKs were consecutively performed using the traditional surgical microscope, OPMI-Lumera 700 (Carl Zeiss Meditec, Inc., Jena, Germany) (TM group).

This study followed the tenets of the Declaration of Helsinki. Informed consent was obtained from all subjects involved in the study.

Forty patients were included in this study. Inclusion criteria for recipient patients comprised age more than 18 years, uneventful previous cataract surgery at least 3 months before DMEK surgery, endothelial corneal dysfunction from FECD and good candidates for lamellar endothelial transplantation. Only cases with sufficient clinical data at 1, 3 and 6 months were included into the study. Exclusion criteria comprised a history of previous ocular surgery (except for cataract surgery), clinically significant posterior capsular opacity, stromal dystrophies, keratoconus, aphakia, history of ocular trauma, glaucoma, active vascular retinal disease, uveitis, myopia more than 6D, age-related macular degeneration and amblyopia.

### 2.2. Materials

**Figure 1:**
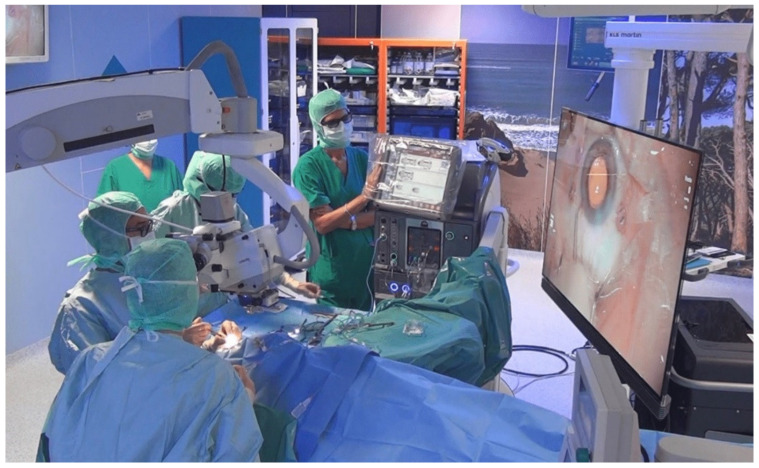
The surgeon operating with a “heads-up” position looking at the 3D monitor with the use of polarized glasses.

Alcon NGENUITY is a modular system that consists of a mobile workstation and an Image Capture Module (ICM), a high-definition stereoscopic 3D image capture camera that is mounted on a standard surgical microscope [15]. The ICM collects light from the microscope and generates a stereoscopic image. The stereoscopic images and videos are sent to a 55-inch 3D high-definition (HD) monitor positioned 1.5 m from the surgeon and arranged perpendicular to the direction of his or her gaze. The resolution of the screen is 4K with a 16:9 format. Figure 1 shows the surgeon operating while looking directly at the monitor with the help of special 3D glasses with passive polarization for stereopsis. This allows them to assume the “heads-up” position, in which the head is raised in a neutral position [16].

The following preoperative donor graft data were collected: donor age (years) and gender, graft endothelial cell density (ECD) (cells/mm^2^) measured by Perseus automated endothelial microscopy (CSO Costruzione Strumenti Oftalmici, Florence, Italy), graft thickness (µm) measured by anterior segment OCT Visante (Carl Zeiss Meditech, Dublin, CA, USA) and preservation time until surgery (days).

The following data were collected preoperatively (baseline), and at 1, 3 and 6 months postoperatively: best spectacle-corrected visual acuity (BSCVA), intraocular pressure (IOP) measured by applanation tonometry (Goldmann applanation tonometer, Haag Streit, Bern, Switzerland), central corneal thickness (CCT) obtained by Anterior Segment OCT MS-39 (CSO Costruzione Strumenti Oftalmici, Florence, Italy), endothelial cell density (ECD) measured by Perseus automated endothelial microscopy (CSO Costruzione Strumenti Oftalmici, Florence, Italy), slit lamp biomicroscopy and ocular fundus examination.

Preoperative and postoperative corneal densitometry (CD) values were assessed by a Pentacam device (Oculus GmbH, Wetzlar, Germany). Scheimpflug CD measures the backscattered light in different concentric regions and layers of the cornea. The light scatter is expressed in grayscale units (GSUs) ranging from 0 GSU, which indicates the maximum corneal transparency, to 100 GSUs, which indicates the minimum corneal transparency [17]. CD was performed for total layer (TL, which includes all the corneal layers) at different annular concentric zones: 0–2 mm zone, 2–6 mm zone and 6–10 mm zone. The peripheral 10–12 mm zone was not included in the study because it was, in all cases, beyond the donor grafts’ diameter. Moreover, intraoperative and postoperative complications including graft unscrolling failure, primary graft failure, graft rejection, Descemet membrane detachments requiring rebubbling and acute IOP decompensation were recorded.

### 2.3. Surgery

All DMEK procedures were performed by the same experienced surgeon (R.M.), who received a 2-month training period for 3D-assisted DMEK surgery, performing at least 15 cases before the beginning of this study. In our study, DMEK procedures were performed under locoregional anesthesia with peribulbar block. Pre-cut DMEK grafts were provided by the Eye Bank of Lucca (Italy), after being stripped and placed on their sclerocorneal support. Grafts were immersed in 0.06% trypan blue dye (Vision blue; D.O.R.C, Zuidland, The Netherlands) and trephined by the surgeon to the preferred width by using a Hessburg-Barron donor corneal punch (Barron Precision Instruments, LLC, Grand Blanc, MI, USA).

The recipient’s cornea was marked with a trephine to guide the subsequent descemetorhexis and to allow the correct positioning of the graft. A clear corneal incision was made to position an anterior chamber maintainer. Descemetorhexis was performed for the central 8.5 to 9 mm diameter using the inverted Price-Sinskey hook, along the epithelial reference line. The removed flap was positioned on the anterior surface of the recipient’s cornea to check its integrity. The pre-cut DMEK graft was carefully detached from the surrounding stroma, immersed in sterile balanced salt solution and aspirated into the glass cartridge of a specific injector (E. Janach S.R.L., Como, Italy). The rolled donor graft was slowly introduced into the recipient’s AC through the main incision. The graft was then unfolded and correctly positioned using the Dirisamer technique. Finally, an air bubble was injected in the AC to press the graft against the recipient’s stroma.

Patients were discharged the same day of surgery and were instructed to keep a supine position until the air bubble was completely reabsorbed. In the case of ocular hypertension or pupillary block, a small quantity of air was released at the slit lamp. The postoperative management for both groups included topical antibiotics given 4 times a day for the first 2 weeks and dexamethasone eye drops 4 times a day for the first month, which was then incrementally reduced over a 6-month period.

### 2.4. Statistics

Statistical analysis was performed using SPSS software (V.28.0 for Windows; IBM SPSS, Chicago, IL, USA). Normality of data distribution in both groups was assessed with the Shapiro–Wilk test. Student’s t-test was used for the following interval scale parameters: BSCVA, IOP, CCT and CD values. Meanwhile, the Mann–Whitney U test was used for ECD. The χ^2^ test was used for categorical variables such as rebubbling rate, intra- and postoperative complication rate. The level of significance was characterized as *p* < 0.05.

## 3. Results

A total of 40 eyes of 40 patients with a median age of 71.5 years were included in this study.

Patient demographics and corresponding preoperative clinical data are shown in Table 1. No significant differences were found between the two groups (*p* > 0.05).

Donor graft data as provided by the eye bank are shown in Table 2. They were similar between the two groups (*p* > 0.05).

Surgical times are shown in Table 3.

In the 3D group, the total surgical time (24.33 min ± 3.56 min vs. 22.01 min ± 3.58 min, *p* = 0.04) and time to perform descemetorhexis (5.25 min ± 2.03 min vs. 3.86 min ± 1.59 min, *p* = 0.02) were significantly higher than in the TM group, while the graft unfolding time was similar between the two groups (4.88 min ± 1.38 min vs. 4.31 min ± 1.31 min, *p* = 0.19).

All clinical outcome parameters at all follow-ups are shown in Table 4 for each study group.

Postoperative results for IOP at all follow-ups were similar in both groups (*p* > 0.05). BSCVA in the 3D group was 0.19 ± 0.14 logMAR at 6 months postoperatively, with no significant differences compared with the TM group (0.19 ± 0.15, *p* = 0.91). The mean CCT in the 3D group was 516.60 ± 23.65 µm at 6 months postoperatively, while in the TM group, it was 515.05 ± 32.88 µm, with no significant difference (*p* = 0.86). Endothelial cell density at 6 months postoperatively was 1639.40 ± 268.31 cells/mm2 in the 3D group (ECD loss rate of 38.5%) and 1654.90 ± 250.76 cells/mm^2^ in the TM group (ECD loss rate of 39.7%), with no significant difference found (*p* = 0.85).

Table 5 shows the preoperative and postoperative total layer (TL) corneal densitometry (CD) for the different concentric zones. Baseline values in the 3D group were 38.91 ± 9.13 GSU for the 0–2 mm zone, 36.05 ± 77.97 GSU for the 2–6 mm zone and 35.9 ± 5.11 GSU for the 6–10 mm zone, while in the TM group, they were 39.79 ± 10.64 GSU, 37.4 ±6.66 GSU and 36.8 ± 4.27 GSU, respectively, with no statistically significant difference (*p* = 0.78, *p* = 0.56 and *p* = 0.55, respectively). Baseline values showed a significant reduction at 6 months in the 3D group (TL CD values were 20.97 ± 4.39 GSU for the 0–2 mm zone, 21.55 ± 2.58 GSU for the 2–6 mm zone and 23.90 ± 3.52 GSU for the 6–10 mm zone) and in the TM group (TL CD values were 19.92 ± 3.86 GSU for the 0–2 mm zone, 19.85 ± 2.05 GSU for the 2–6 mm zone and 25.30 ± 2.67 GSU for the 6–10 mm zone), showing no statistically significant difference between the two groups (*p* = 0.42, *p* = 0.12 and *p* = 0.16, respectively).

Rebubbling occurred in four cases in both groups (20% rebubbling rate in 3D and TM groups) within the first month, with an uneventful postoperative course. One case of acute IOP decompensation that required air deflation within the first 24 h was recorded in the TM group. No regrafting was needed for both groups.

## 4. Discussion

DMEK surgery represents one of the most successful treatment options for endothelial disease because of the fast visual recovery combined with the very low incidence of graft failure and graft rejection compared with penetrating keratoplasty [18].

Considering the very low thickness of the graft, it is crucial for the surgeon to have optimal intraoperative visibility and surgical comfort. Consequently, 3D visualization systems may provide an intraoperative detailed view and better surgical ergonomics, thus reducing physical strain, which is known to be widely prevalent among surgeons in ophthalmology [19].

This is, to the best of our knowledge, the first study to investigate the clinical outcomes, including corneal densitometric values, of DMEK surgery in pseudophakic patients with FECD using a 3D system versus a traditional microscope with a 6-month follow-up.

Only a few other studies have investigated the use of 3D visualization systems in corneal transplantation surgery, most of them being case reports [11,12,13,20]. Mohamed YH et al. reported the first case of corneal surgery using a heads-up system [11]. They performed non-Descemet Stripping Automated Endothelial Keratoplasty (nDSAEK) using 3D technology for a post-traumatic bullous keratopathy and reported a great visual and ergonomic experience. However, the authors stated that frequent focus adjustment was required for a clear stereoscopic view of the flap.

Panthier et al. showed, in a prospective study, the outcomes 3 months after DMEK surgery performed using the NGENUITY 3D visualization system versus a traditional microscope in 24 patients with FECD and pseudophakic bullous keratopathy [13]. The authors found no significant differences in clinical outcomes in the two groups despite the longer surgical times of the 3D group. Nevertheless, they included single DMEK procedures and triple procedures (DMEK combined with phacoemulsification and posterior chamber lens implantation) [13].

The present retrospective study of 40 eyes that underwent DMEK surgery with either a 3D visualization system (*n* = 20) or traditional surgical microscope (*n* = 20) showed a significantly longer global surgical time (*p* = 0.04) and a significantly longer time to perform descemetorhexis (*p* = 0.03) in the 3D group. Nonetheless, the longer surgical time of the 3D group was not crucial as the planned sequence of surgeries of the operating session was not affected in any case.

Conversely, similar outcomes for BSCVA, ECD and CCT values at all follow-ups (*p* > 0.05) could be detected in the 3D group and TM group. We also recorded Scheimpflug CD as an objective parameter for assessing corneal transparency. According to our knowledge, only a few studies analyzing CD after DMEK surgery have been published [5,6,21]. We observed a reduction in CD values in the 6-month follow-up period, with no significant difference in the two groups, implying a similar improvement in corneal clarity after DMEK. Moreover, intraoperative and postoperative complication rates, such as acute IOP decompensation, graft failure and graft rejection rates, were similar between the two groups. Significant graft detachment requiring a rebubbling procedure after DMEK surgery was observed with the same rate of 20% in both groups.

According to our results, 3D-assisted DMEK surgery provided similar outcomes in terms of efficacy and safety compared with DMEK cases performed with a conventional microscope over a 6-month follow-up period.

Furthermore, the surgeon reported better intraoperative ergonomics allowing for a greater degree of freedom during surgery, despite a subtle latency effect (70 ms) due to the processing time of the Image Capture Module, which, nevertheless, did not affect the fluency of the surgeon’s maneuvers [22].

The major benefits of a heads-up approach in ophthalmic surgery are described in the literature and they include the more ergonomic position of the operator, the excellent teaching capacity of the 3D image, which is shared in the operating room among all the staff, a wider visual field with a greater image resolution and the possibility to apply digital filters to the 3D image projected on the screen [23,24,25].

The limitations of our study were the retrospective non-randomized design, the involvement of a relatively small number of patients, as well as the involvement of a single surgeon performing DMEKs. Further multicentric, prospective, randomized studies are warranted to assess the outcomes of heads-up DMEK compared to the surgery performed with the TM.

Moreover, since our study excluded patients with concomitant corneal disorders, complex anterior segment anatomy or a history of previous corneal surgery, we could not investigate the efficacy and safety of 3D-assisted DMEK surgery in complex cases.

In conclusion, we believe that a heads-up approach can be employed to assist DMEK surgery, providing good results in terms of clinical outcomes, despite a slightly longer surgical time. The outstanding teaching capacity as well as the improved comfort provided by the heads-up approach may encourage anterior segment surgeons to implement this new technology for their routine keratoplasty cases, especially in teaching hospitals.

## Figures and Tables

**Table 1:** Patient demographics and corresponding preoperative clinical data.

	3D Group		TM Group		
	Mean ± SD (Range)	Absolute Number (%)	Mean ± SD (Range)	Absolute Number (%)	*p* Value
**Age (years)**	72.6 ± 6.9 (58–87)		68.6 ± 7.4 (54–85)		0.09
**Male**		7 (35%)		9 (45%)	
**Female**		13 (65%)		11 (55%)	0.42
**Right Eye**		9 (45%)		8 (40%)	
**Left Eye**		11 (55%)		12 (60%)	0.85
**BSCVA** **(logMAR)**	0.43 ± 0.21 (0.20–0.90)		0.54 ± 0.42 (0.20–2.00)		0.31
**CCT (µm)**	641.25 ± 50.2 (585–750)		643.05 ± 46.62 (598–766)		0.90
**IOP (mmHg)**	14.4 ± 1.4 (12–17)		13.9 ± 1.4 (10–16)		

BSCVA, best spectacle-corrected visual acuity; CCT, central corneal thickness; IOP, intraocular pressure; logMAR, logarithm of the minimum angle of resolution.

**Table 2:** Donor graft data.

	3D Group		TM Group		
	Mean ± SD (Range)	Absolute Number (%)	Mean ± SD (Range)	Absolute Number (%)	*p* Value
**Age (years)**	68.7 ± 7.2 (54–76)		64.9 ± 6.8 (45–74)		0.10
**Male**		7 (35%)		9 (45%)	
**Female**		13 (65%)		11 (55%)	
**ECD (cells/mm^2^)**	2667.5 ± 192.1 (2300–3000)		2742.8 ± 136.9 (2500–3000)		0.16
**Preservation time (days)**	27.2 ± 3.3 (22–36)		26.2 ± 2.4 (21–30)		0.30

ECD, endothelial cell density.

**Table 3:** Surgical times.

	3D Group	TM Group	
	Mean ± SD (Range)	Mean ± SD (Range)	*p* Value
**Total surgical time (min)**	24.33 ± 3.56 (17.80–29.60)	22.01 ± 3.58 (15.40–30.40)	0.04
**Time to perform descemetorhexis (min)**	5.25 ± 2.03 (1.20–8.20)	3.86 ± 1.59 (0.90–7.20)	0.02
**Graft unfolding time (min)**	4.88 ± 1.38 (2.80–7.20)	4.31 ± 1.31 (2.50–6.90)	0.19

**Table 4:** Patient postoperative clinical data.

	Time	3D Group	TM Group	*p* Value
		Mean ± SD (Range)	Mean ± SD (Range)	
**BSCVA (logMAR)**	1° month	0.33 ± 0.25 (0.1–1.0)	0.29 ± 0.21 (0.1–1.0)	0.68
	3° month	0.22 ± 0.17 (0–0.6)	0.21 ± 0.19 (0–0.8)	0.86
	6° month	0.20 ± 0.14 (0–0.4)	0.19 ± 0.16 (0–0.6)	0.92
**CCT (µm)**	1° month	543.1 ± 35.69 (515–657)	542.95 ± 34.99 (520–680)	0.99
	3° month	525.15 ± 20.56 (505–603)	516.95 ± 25.21 (490–600)	0.27
	6° month	516.6 ± 23.66 (495–603)	515.05 ± 32.88 (487–611)	0.87
**ECD (cells/mm^2^)**	1° month	1787.6 ± 300.13 (1250–2302)	1815.9 ± 220.66 (1470–2384)	0.74
	3° month	1711.15 ± 282.87 (1200–2200)	1712.05 ± 197.83 (1300–2200)	0.99
	6° month	1639.4 ± 268.32 (1200–2130)	1654.9 ± 250.76 (1359–2280)	0.85

BSCVA, best spectacle-corrected visual acuity; CCT, central corneal thickness; ECD, endothelial cell density; logMAR, logarithm of the minimum angle of resolution.

**Table 5:** Patient preoperative and postoperative corneal densitometry.

		3D Group	TM Group	
	Time	Mean ± SD (Range)	Mean ± SD (Range)	*p* Value
**CD 0–2 mm (GSU)**	Baseline	38.91 ± 9.13 (25.7–55.8)	39.79 ± 10.64 (29.0–79.5)	0.78
	1° month	24.53 ± 6.03 (16.0–39.0)	23.03 ± 2.40 (19.0–27.0)	0.40
	3° month	22.78 ± 3.81 (16.0–30.0)	22.38 ± 4.05 (16.0–29.0)	0.75
	6° month	20.98 ± 4.39 (14.0–29.0)	19.93 ± 3.86 (14.0–27.0)	0.43
**CD 2–6 mm (GSU)**	Baseline	36.05 ± 77.97 (28.0–65.2)	37.4 ±6.66 (30.2–62.3)	0.56
	1° month	23.1 ± 3.7 (19.0–33.0)	21.85 ±2.87 (17.0–19.0)	0.24
	3° month	21.88 ± 2.73 (18.0–28.0)	20.9 ± 2.13 (16.0–25.0)	0.27
	6° month	20.70 ± 2.16 (17.0–25.0)	19.85 ± 2.06 (16.0–24.0)	0.22
**CD 6–10 mm (GSU)**	Baseline	35.9 ± 5.11 (29.1–45.0)	36.8 ± 4.27 (32.1–44.8)	0.55
	1° month	25.8 ± 4.94 (18.0–37.0)	25.4 ± 2.35 (21.0–29.0)	0.75
	3° month	24.55 ± 3.95 (17.0–33.0)	25.6 ± 2.7 (21.0–31.0)	0.33
	6° month	23.9 ± 3.53 (16.0–30.0)	25.3 ± 2.68 (21.0–30.0)	0.17

CD, corneal densitometry; GSU, grayscale units.

## Data Availability

Data not publicly available.

## References

[1] Nanavaty M.A., Wang X., Shortt A.J. (2014). Endothelial keratoplasty versus penetrating keratoplasty for Fuchs endothelial dystrophy. Cochrane Database Syst. Rev..

[2] Ang M., Wilkins M.R., Mehta J.S., Tan D. (2016). Descemet membrane endothelial keratoplasty. Br. J. Ophthalmol..

[3] Waldrop W.H., Gillings M.J., Robertson D.M., Petroll W.M., Mootha V.V. (2020). Lower Corneal Haze and Aberrations in Descemet Membrane Endothelial Keratoplasty Versus Descemet Stripping Automated Endothelial Keratoplasty in Fellow Eyes for Fuchs Endothelial Corneal Dystrophy. Cornea.

[4] Pahuja N., Shetty R., Subbiah P., Nagaraja H., Nuijts R.M., Jayadev C. (2016). Corneal Densitometry: Repeatability in Eyes with Keratoconus and Postcollagen Cross-Linking. Cornea.

[5] Alnawaiseh M., Zumhagen L., Wirths G., Eveslage M., Eter N., Rosentreter A. (2016). Corneal Densitometry, Central Corneal Thickness, and Corneal Central-to Peripheral Thickness Ratio in Patients with Fuchs Endothelial Dystrophy. Cornea.

[6] Schaub F., Enders P., Bluhm C., Bachmann B.O., Cursiefen C., Heindl L.M. (2017). Two-Year Course of Corneal Densitometry after Descemet Membrane Endothelial Keratoplasty. Am. J. Ophthalmol..

[7] Eckardt C., Paulo E.B. (2016). Heads-up surgery for vitreoretinal procedures: An experimental and clinical study. Retina.

[8] Kunikata H., Abe T., Nakazawa T. (2016). Heads-up macular surgery with a 27-gauge microincision vitrectomy system and minimal illumination. Case Rep. Ophthalmol..

[9] Skinner C.C., Riemann C.D. (2018). ‘Heads up’ digitally assisted surgical viewing for retinal detachment repair in a patient with severe kyphosis. Retin. Cases Brief Rep..

[10] Coppola M., La Spina C., Rabiolo A., Querques G., Bandello F. (2017). Heads-up 3D vision system for retinal detachment surgery. Int. J. Retina. Vitr..

[11] Mohamed Y.H., Uematsu M., Inoue D., Kitaoka T. (2017). First experience of nDASEK with heads-up surgery. Medicine.

[12] Galvis V., Berrospi R.D., Arias J.D., Tello A., Bernal J.C. (2017). Heads up Descemet membrane endothelial keratoplasty performed using a 3D visualization system. J. Surg. Case Rep..

[13] Panthier C., Courtin R., Moran S., Gatinel D. (2021). Heads-up Descemet Membrane Endothelial Keratoplasty Surgery: Feasibility, Surgical Duration, Complication Rates, and Comparison with a Conventional Microscope. Cornea.

[14] Weinstock R.J., Diakonis V.F., Schwartz A.J., Weinstock A.J. (2019). Heads-up cataract surgery: Complication rates, surgical duration, and comparison with traditional microscopes. J. Refract. Surg..

[15] Moura-Coelho N., Henriques J., Nascimento J., Dutra-Medeiros M. (2019). Three-dimensional Display Systems in Ophthalmic Surgery—A Review. Eur. Ophthalmic. Rev..

[16] Berquet F., Henry A., Barbe C., Cheny T., Afriat M., Benyelles A.K., Bartolomeu D., Arndt C. (2020). Comparing Heads-Up versus Binocular Microscope Visualization Systems in Anterior and Posterior Segment Surgeries: A Retrospective Study. Ophthalmologica.

[17] Dhubhghaill S.N., Rozema J.J., Jongenelen S., Hidalgo I.R., Zakaria N., Tassignon M.J. (2014). Normative values for corneal densitometry analysis by Scheimpflug optical assessment. Investig. Ophthalmol. Vis. Sci..

[18] Ong H.S., Ang M., Mehta J. (2021). Evolution of therapies for the corneal endothelium: Past, present and future approaches. Br. J. Ophthalmol..

[19] Weinstock R.J., Ainslie-Garcia M.H., Ferko N.C., Qadeer R.A., Morris L.P., Cheng H., Ehlers J.P. (2021). Comparative Assessment of Ergonomic Experience with Heads-Up Display and Conventional Surgical Microscope in the Operating Room. Clin. Ophthalmol..

[20] Borroni D., Rocha-de-Lossada C., Bonci P., Rechichi M., Rodríguez-Calvo-de-Mora M., Rachwani-Anil R., Sánchez González J.M., Urbinati F., Lorente M.G., Vigo L. (2022). Glasses-Assisted 3D Display System-Guided Descemet Membrane Endothelial Keratoplasty Tissue Preparation. Cornea.

[21] Agha B., Dawson D.G., Kohnen T., Schmack I. (2019). Corneal Densitometry after Secondary Descemet Membrane Endothelial Keratoplasty. Cornea.

[22] Ta Kim D., Chow D. (2022). The effect of latency on surgical performance and usability in a three-dimensional heads-up display visualization system for vitreoretinal surgery. Graefes. Arch. Clin. Exp. Ophthalmol..

[23] Del Turco C., D’Amico Ricci G., Dal Vecchio M., Bogetto C., Panico E., Giobbio D.C., Romano M.R., Panico C., La Spina C. (2022). Heads-up 3D eye surgery: Safety outcomes and technological review after 2 years of day-to-day use. Eur. J. Ophthalmol..

[24] Mendez B.M., Chiodo M.V., Vandevender D., Patel P.A. (2016). Heads-up 3D Microscopy: An Ergonomic and Educational Approach to Microsurgery. Plast. Reconstr. Surg. Glob. Open..

[25] Bin Helayel H., Al-Mazidi S., AlAkeely A. (2021). Can the Three-Dimensional Heads-Up Display Improve Ergonomics, Surgical Performance, and Ophthalmology Training Compared to Conventional Microscopy?. Clin. Ophthalmol..

